# A General System for Automatic Biomedical Image Segmentation Using Intensity Neighborhoods

**DOI:** 10.1155/2011/606857

**Published:** 2011-06-23

**Authors:** Cheng Chen, John A. Ozolek, Wei Wang, Gustavo K. Rohde

**Affiliations:** ^1^Department of Biomedical Engineering, Center for Bioimage Informatics, Carnegie Mellon University, Pittsburgh, PA 15213, USA; ^2^Department of Pathology, Children's Hospital of Pittsburgh, Pittsburgh, PA 15224, USA; ^3^Department of Electrical and Computer Engineering, Carnegie Mellon University, Pittsburgh, PA 15213, USA; ^4^Lane Center for Computational Biology, Carnegie Mellon University, Pittsburgh, PA 15213, USA

## Abstract

Image segmentation is important with applications to several problems in biology and medicine. While extensively researched, generally, current segmentation methods perform adequately in the applications for which they were designed, but often require extensive modifications or calibrations before being used in a different application. We describe an approach that, with few modifications, can be used in a variety of image segmentation problems. The approach is based on a supervised learning strategy that utilizes intensity neighborhoods to assign each pixel in a test image its correct class based on training data. We describe methods for modeling rotations and variations in scales as well as a subset selection for training the classifiers. We show that the performance of our approach in tissue segmentation tasks in magnetic resonance and histopathology microscopy images, as well as nuclei segmentation from fluorescence microscopy images, is similar to or better than several algorithms specifically designed for each of these applications.

## 1. Introduction

In the past few decades, we have witnessed a great increase in the development of new imaging modalities, and their applications to different biomedical research and clinical problems. Given the enormous success with which some of these technologies have been applied to research and clinical tasks, the trend of image technology development (and its application in novel biomedical problems) is likely to continue. Segmentation is of great importance in the application of imaging technology to many biomedical problems. Tissues, cells, and organs must often be segmented and isolated from two- or three-dimensional digital image data for subsequent quantitative analysis in a variety of experimental biological studies and diagnosis in clinical medicine. Popular examples include quantification of gray and white matter tissues from magnetic resonance imaging (MRI) brain scans for studying neurological diseases (e.g., Alzheimer's), and segmentation of cells and tissues from histopathology image data to assist in the diagnosis of different lesions, segmentation of cells and subcellular structures for characterizing their distribution, to name a few.

Due to the vast increase in the capability of image acquisition during the past couple of decades, manual segmentation is no longer a viable option for many types of quantitative studies. The increase in computational power in recent decades has spurred the development of several image segmentation algorithms (see [1–8] for reviews on this topic) that have had a significant impact in several clinical and research applications. It is worth noting, however, that many algorithms successfully used in real applications were specifically designed for the given application. In our previous experience, before an algorithm that was specifically designed for one application can be used in another, a significant amount of tuning and calibration is usually required [1, 3, 5]. Even then, in many cases, the chosen method may not perform satisfactorily. Therefore, a researcher faced with a new problem or application must often spend considerable resources to modify (or develop anew) a reliable segmentation method capable of extracting the structures of interest for the given application.

Amongst the several currently available alternatives for image segmentation, methods based on pixel classification utilizing learning-based classification strategies are attractive because, in principle and given enough training samples, the strategy can be used to construct algorithms capable of performing accurately across different image modalities (computed tomography (CT), MRI, microscopy, e.g.,) and different structures of interest (organs, tissues, or cells). Several algorithms based on this strategy have been described. We mention a few recent ones, focused on biomedical image segmentation applications for different modalities of data: Debeir et al. [9] used statistical features from color channels, together with a pixel classification by decision tree method, to segment pigmented skin lesions in epiluminescence microscopy (ELM) images. Madabhushi et al. [10] described a pixel level classification system that utilized 3D texture features to segment the malignant regions in MR prostatic images for diagnosis of prostatic adenocarcinoma. In [11], Bhagavatula et al. used a histopathology vocabulary (HV) features and pixel classification strategy to identify germ-layer components in hematoxylin and eosin (H&E) stained histological images of teratomas. Considering that segmentation methods based on pixel classification are often computationally inefficient, Dam and Loog [12] proposed a general sparse pixel classification method. While reasonably high accuracies can be obtained with the methods mentioned above, to our best knowledge, no general purpose approach for segmenting different structures (cells or tissue types) in different imaging modalities has been proposed and shown to work well across different applications. We believe that, in part, this is due to the fact that different applications, structures, and imaging modalities require careful selection of relevant parametric features to work well.

Here, we describe a system for biomedical image segmentation based on pixel classification using intensity neighborhoods. While intensity neighborhoods have been used to classify texture patches before (several works are discussed in the following section), we show here that such an approach (with adaptations) can be used as a general segmentation tool for biomedical images. The system is general in the sense that, given sufficient training data, it can be used to segment a variety of biological structures, from different imaging modalities. In addition, the algorithm can be used with two- or three-dimensional data, as well as scalar or vector valued (e.g., color) images. Our algorithm requires as input a few already segmented images/structures (often obtained manually) with which a classifier is trained. In order to arrive at a generic segmentation/classification method, with each pixel in the training set, we associate a nonparametric feature vector consisting of the neighborhood intensities for building the classification-based segmentation system. The system aims to maximize the available training data by exploring several orientations as well as scales for each labeled pixel. We compare the performance of our general image segmentation method to several state-of-the-art methods for segmenting brain MR images, histopathological images, and fluorescence images of nuclei. We show that, using the methodology we describe in detail below, such a generic segmentation system can achieve accurate segmentation results in a variety of applications.

## 2. Methods

Our algorithm aims to make use of the fact that often times a few segmented images (labeled pixel data) are already available or can be easily obtained, together with the ever increasing capabilities of modern computers, to arrive at a general segmentation tool that can be used in a variety of applications. This can be achieved within a supervised learning strategy where the pixels of a few segmented images are used to train a classifier capable of accurately determining the class (e.g., background versus organs/tissues of interest) of each pixel in unlabeled images of the same kind. For the method to work well across several applications, it is important that the information present in the training set be appropriately used during training of the classifier. By imposing a predefined size window centered at the given pixel, in which pixels inside the window are regarded as neighbors, the intensity neighborhood vectors are thus constructed by reordering the pixels' intensities inside the window into a vector and used instead of parametric feature vectors in order to allow the method to be general. This, however, requires that the data be appropriately normalized and that different variations such as scales, rotations, and so forth be taken into account. In its simplest form, such a classifier would need to be trained utilizing all available pixels as training data (accounting for normalization), while different variations and scales of the same data (which are simulated as described below) will also need to be taken into account. This strategy, when applied to its full extent, would be computationally impractical. For example, the support vector machine classifier we select to use in our implementation has a computational cost of order *P*^3^ (upper bound) [13], with *P* the number of pixels. Since very often millions of pixels are available for training, it is clear a different approach must be taken.

**Figure 1:**
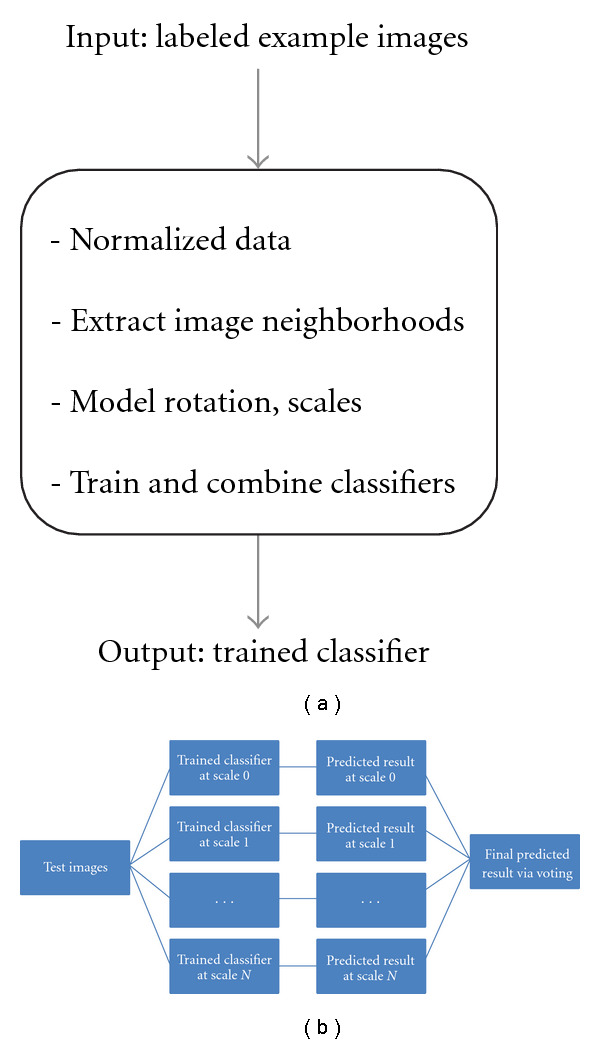
System schematic organization for neighborhood-based image segmentation using supervised learning (a), training stage (b), and testing stage.

In this paper, we propose an empirical approach for reducing the amount of training data, while retaining important information that allows one to differentiate between pixel classes, thus allowing for execution in reasonable computation time. The strategy is conceptually simple (it is summarized in Figure 1), and we show it works well in several practical applications. In our algorithm, the training stage occurs by first normalizing the input data, extracting important pixels from the input data, modeling rotation and scalings of the data via filtering and resampling, and training the actual classifiers at different scales (Figure 1(a)). In the testing stage, the final result is given through a voting procedure for combining the predictions under different scales (shown in Figure 1(b)). We also note that several of the substeps in our procedure (e.g., multiscale filters, classification method, etc.) may be replaced by other options, in the presence of more detailed application-specific information.

### 2.1. Intensity Normalization

Given an application (e.g., segmentation of tissues in histopathological images), it is important to account for variations in intensity (e.g., due to images being taken with different microscopes under different illumination environments, etc.) that may be present from image to image. In MRI, for example, intensity normalization for correction of different bias fields is an extensively studied subject [14, 15]. The calibration for intensity in histopathological images, or fluorescent images can also be performed [16], in limited controlled settings, however. Since our aim is to design a system for general purposes, we do not rely on intensity corrections specifically designed for any single-imaging modality (or application) but rather take the general approach of normalizing all image data to fit the intensity range of [0,1] by scaling the minimum and maximum of each image (discounting outliers set at 1% in our implementation). We note, however, that given more detailed imaging/application-specific information, this method can be replaced by other alternatives.

### 2.2. Intensity Neighborhood Statistics: What Information Should Be Considered?

Instead of parametric features (e.g., Haralick texture features, Gabor filters, etc.), we select each pixel's neighborhood intensities as a general nonparametric feature set for classification. When a large enough window is chosen, the advantage of such an approach is that all information relevant for differentiating different classes (tissues, cells, etc.) will be preserved. The disadvantages are that, especially if large neighborhoods are necessary, the method will involve classification in high-dimensional spaces, often making accurate estimations difficult. The idea of utilizing window neighborhoods is not novel, and has been used in numerous applications in image processing. For example, Varma and Zisserman [17] describe a classification method based on pixels' neighborhood intensities. They showed the method compared favorably to the more traditional parametric filter-bank method on an application related to texture segmentation. Awate et al. also present the neighborhood based method for texture segmentation [18] and brain-MR segmentation [19]. In addition, similar neighborhood ideas have been used in image filtering and denoising fields recently. Buades et al. introduced the non-local means (NLM) method [20] for image denoising, in which similarities of image neighborhood are calculated as weights for averaging pixel intensities. In [19], this neighborhood weighted averaging method is interpreted statistically. In order to reduce the computational cost caused by the high dimensionality of neighborhood in NLM method, principal component analysis (PCA) of neighborhood is used in NLM method [21]. We note, however, that although neighborhood-based image segmentation methods have been described in the past, to our knowledge, no general algorithm has been proposed, validated, and compared to state of the art techniques in several different biomedical image segmentation problems (including different imaging modalities). Here, we describe several adaptations and modifications of the nonparametric intensity neighborhood idea to arrive at a generic image segmentation system capable of performing well across a variety of biomedical applications.

**Figure 2:**
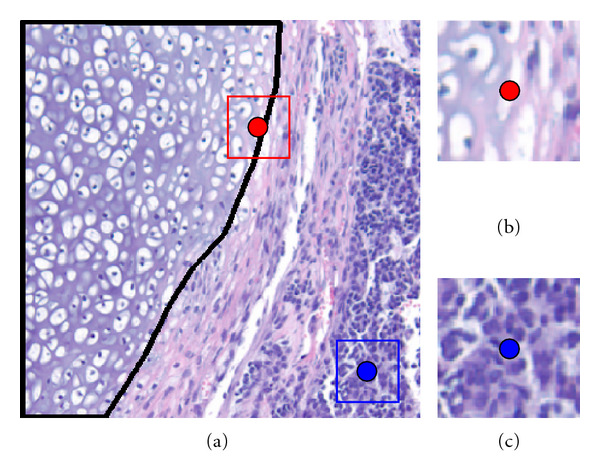
Examples of Boundary Type Pixel and Interior Type Pixel. (a) Original image with two pixels marked. (b) Magnified window of boundary-type pixel. (c) Magnified window of interior-type pixel. Black line indicates boundary between tissue types.

In our implementation, we utilize a *N* × *N* square patch for 2D data (and a *N* × *N* × *N* cube patch for 3D data), to comprise the *N*^2^ (or *N*^3^) nonparametric feature vector associated with the pixel at the center of the patch (*N* is chosen to be an odd number in our application). Since the image patches are not rotationally invariant, the training set as described could potentially be suboptimal since it does not include rotated versions of the feature vector while for most biomedical applications of interest, a fixed coordinate frame for the tissues/cells of interest cannot be assumed. To overcome this limitation, we synthetically augment the training set of images by including image patches that are also rotated (about multiple axes if necessary). Image rotation is computed using linear interpolation in our case. Consideration of computational complexity limits us to utilize only a limited number of angles (described in detail below for each application), however. In addition, we include flipped (coordinate reversed) versions of windows to increase the amount of useful information. In order to further reduce the number of potential training pixels, we also make the assumption that it is more important to include variations of patches from pixels near the boundaries between two or more classes (e.g., tissues) than interior pixels, allowing us to consider only multiple rotated and flipped versions of boundary-type pixels' neighborhood patches in our training set, reducing the computational complexity of the procedure. A general way to decide the target pixel's type is to see whether a square window centered at the target pixel includes more than two classes. If so, such pixel will be judged as boundary type pixel, otherwise, it will be judged as interior type pixel. In Figure 2, we illustrate examples of both boundary type pixel and interior type pixel. Figure 2(a) shows the original image, in which the boundary type pixel is represented by a red dot, and interior type pixel is represented by a blue dot. For better understanding, we also show two square windows centered at two pixels. In Figures 2(b) and 2(c), we zoom in the window to show clear tissue appearances around the pixels. Clearly, we can see that the square window patch of boundary type pixel contains two different tissue textures, while the square window of interior type pixel just contains one type of tissue.

Finally, it is also important to consider multiscale information. While for some types of tissues/cells, information for distinguishing a given object from others may be present in relatively small (local) neighborhoods, and for others, the overall architecture (big picture) of the neighborhood is necessary to provide crucial information. More generally, we believe information from multiple scales should be used to characterize each class in each application. Arguably, this could be achieved by simply considering the size of the neighborhood (*N*) to be large enough and leave it to a classifier to determine the critical information for each class. However, large neighborhoods have the disadvantage that they amount to high-dimensional spaces, making estimation and pixel classification difficult. In order to utilize small neighborhoods (e.g., *N* = 3 or 5) while being able to capture the useful information for different classes, we use a standard multiresolution sequence [22]. In short, *N* remains constant for each scale. At each scale, images belonging to the training set are first convolved with a Gaussian kernel for smoothing, and sampled (according to the scale chosen) to construct the neighborhood set associated with a particular scale. More specifically, given scales *s* ∈ {0,1, 2,…, *S*}, neighborhood patches are assembled by subsampling at every 2^*s*^ pixels (after smoothing). Therefore, we can associate each pixel in the training set (as well as during actual classification of a test pixel) several sets of neighborhoods with the same size *N* but under different resolutions, comprising the multiple scales associated with that pixel.

### 2.3. Algorithm for Selecting Representative Pixel Neighborhoods

Our system works by utilizing a set of pixels from pre-segmented images to train a classifier and then segmenting new unseen images. For many applications, however, even relatively few images (when the variations in rotation and scale are included) can contain several million pixels in the training samples. Given the computational complexity of pertinent classifiers, which generally ranges from *O*(*P*^2^) to *O*(*P*^3^), with *P* the number of training samples [2, 13], simply utilizing all available pixels for training is not a practical strategy. Given the maximum number of training samples (denoted as *Q* and preselected in order to satisfy computational cost considerations) to be utilized for training, one alternative for selecting such *Q* pixels would be to do so at random. Here, we describe an alternative procedure based on the *K*-means algorithm for selecting a set of training pixels that retains the main “trends” of the information contained in the training pixels.

We note that since the computational complexity of the *K*-means procedure is also of *O*(*P*^2^), its straightforward application is difficult. We, therefore, resort to the following simplification. We first divide the *P* training samples into different subsets (in this paper, the subsets are divided according to their spatial location in the image). We then apply the *K*-means method to each subset separately and combine all the clustered samples into a predefined training set of size *Q* samples. The procedure can be summarized as follows.

**Figure 3:**
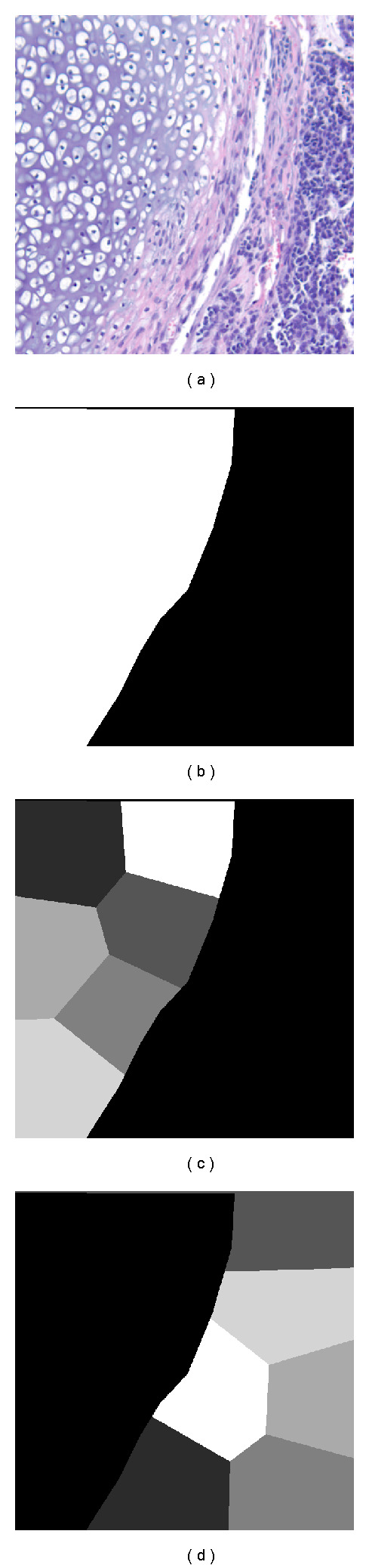
Examples of spatial sample separation. (a) Original image. (b) Ground truth. (c) Spatial sample separation in interested tissue. (d) Spatial sample separation in background/other tissues.

1. We use the *K*-means method to cluster the pixels' spatial coordinates, which divides the input training images into *R* nonoverlapping spatial regions, with *R* selected manually (see Figure 3).
2. Each of the pixels in the regions defined by step (1) are categorized as either a boundary-type pixel or an interior-type pixel. In each region, the *K*-means procedure is used to select only certain pixels (and their windows) for training (the amount selected is calculated based on *R* and *Q*). This is done for both boundary (including rotated and flipped windows) and interior type pixels, and is repeated for each scale *s*.
3. Finally, for each scale *s*, all the clustered samples for both boundary-type and interior-type of all subsets and all classes are combined together as the training set containing *Q* samples and thereby, several training sets associating with different scales *s* ∈ {0,1,…, *S*} can be built.

### 2.4. Algorithm for Classification

We utilize the support vector machine (SVM) classifier [13, 23] as the algorithm for classifying each pixel. In a two-class problem, given a training set containing *Q* samples (each with *d* dimensions), we denote the feature-label pairs as (*X*_*i*_, *Y*_*i*_), *i* = 1,…, *Q*, where *X*_*i*_ ∈ **R**^*d*^, *Y*_*i*_ ∈ {−1, +1}. The support vector machine determines parameters *w* and *b* such that

(1) $$\begin{array}{cc} & \underset{w,b,\xi }{\text{argmin}}\;\left\{\frac{1}{2}w^{T}w+\varphi \overset{Q}{\underset{i=1}{\sum }}\xi_{i}\right\},\\ & \begin{array}{c} \text{subject}\;\;\text{to}\;Y_{i}\left(w^{T}\phi \left(X_{i}\right)+b\right)\geq 1-\xi_{i},\;\xi_{i}\geq 0 \end{array} \end{array}$$

is minimized, yielding a hyperplane (defined by *w* and *b*) that linearly separates the data. Since the dataset is not always linearly separable, the *ξ*_*i*_ represents the distance of each error point *i* to its correct plane, and *φ* is a penalty constant for the error term. *ϕ* is a fixed nonlinear mapping function (known as basis function) that extends training vectors *X*_*i*_ into higher-dimensional space *ϕ*(*X*) : **R**^*d*^ ↦ **R**^*m*^. This problem is usually solved in its dual representation, where the data always occur in pairs, with the aid of the kernel function [23, 24] *K*(*X*_*i*_, *X*_*j*_) = *ϕ*(*X*_*i*_)^*T*^*ϕ*(*X*_*j*_). Common kernel functions include: the linear kernel, the Gaussian radial basis function (RBF) kernel, polynomial kernel, sigmoid kernel, and so forth. In our work, the RBF kernel *K*(*X*_*i*_, *X*_*j*_) = exp (−*γ*||*X*_*i*_−*X*_*j*_||^2^), *γ* ≥ 0 was selected for all applications, because such kernel is able to handle the case when the relation between class labels and attributes is nonlinear by nonlinearly mapping samples into a higher dimensional space. For problems with more than two classes, we use the “one-against-all” classification strategy [25] to reduce the single multiclass problem into multiple binary problems and use a max-wins voting strategy to combine these binary results and classify the testing instance. The classification system was implemented utilizing the LibSVM software [26] package.

The optimal parameters, such as the penalty constant *φ* and the RBF kernel size *γ*, are selected using a cross validation procedure [26]. We use *k*-fold cross-validation to further separate the training set into two parts (lower-level), and search for the parameters (*φ*, *γ*), which have the best accuracy in this *k*-fold cross-validation. We set *k* = 10, and perform an exhaustive search for the two parameters: firstly, we search (for (*φ*, *γ*)) broadly using a large step size in the range which could be considered reasonable (determined empirically). If any optimal parameters are selected on their lower (or upper) bounds of the ranges, we decrease (or increase) the corresponding bounds by 5 times, and repeat the rough (broad) search. If the optimal parameters are selected inside the ranges, we then select smaller ranges around the optimal parameters, and choose a smaller step size to find the final optimal parameters locally. The ranges (or the upper bounds) are first determined empirically. If any optimal parameters are selected on their lower (or upper) bounds of the ranges, we decrease (or increase) the corresponding bounds by 5 times, and repeat the rough search. More details of the descriptions can be seen in [27]. After the optimal parameters are selected, we use them to build the classifiers and evaluate their performance on the testing data.

### 2.5. Multiscale Classifier Ensemble: How to Integrate Information from Individual Classifiers?

As we stated above, we associate each pixel in the training set of images several neighborhoods each containing different scale information (scales *s* ∈ {0,1, 2,…, *S*} defined above). Instead of combining all scales into a single high-dimensional vector (an option we investigated but did not yield adequate segmentation results presumably due to aforementioned difficulties with dealing with high dimensional data), we opt instead to train *S* + 1 classifiers and combine them so that the final overall accuracy is higher than that obtained from any single scale. We note that several methods for combining multiple classifiers exist [28–32], some of which have been applied to the problem of pixel classification using parametric features (see, e.g., [33, 34]). Amongst the most simple, are voting-based methods. In our system, we test two popular voting strategies: majority voting and confidence-based voting.

**Majority Voting**

(2) $$l_{i}^{\text{final}}=\underset{c\in \left\{1,2,\ldots ,C\right\}}{\arg\;\max\;}\;\overset{S}{\underset{s=0}{\sum }}\delta \left(l_{i}^{s},c\right),$$

where *δ*(*i*, *j*) is defined as

(3) $$\delta \left(i,j\right)=\{\begin{array}{cc} 1, & i=j,\\ 0, & i\neq j. \end{array}$$

**Confidence-Based Voting**

In the confidence voting algorithm, classifiers are also trained separately for each scale *s*. After the training procedure, it is often possible to assign a confidence to the assignment of any pixel, denoted as *F*(*l*_*i*_^*s*^ = *c*). In our case, we utilize the posterior probability in SVM: *P*(*l*_*i*_^*s*^ = *c* | **x**_*i*_) (**x**_*i*_ is the intensity neighborhood vector for the given pixel *i*) as the confidence measurement, although other alternative confidence estimates can be used. For multiple classes, the posterior probability in SVM can be estimated by combining all the pairwise class probabilities [35]. The confidence-based voting strategy is then implemented as

(4) $$l_{i}^{\text{final}}=\underset{c\in \left\{1,2,\ldots ,C\right\}}{\arg\;\max\;}\;\overset{S}{\underset{s=0}{\sum }}\lambda_{s}F\left(l_{i}^{s}=c\right).$$

We test and compare two confidence-based voting strategies: weighted and unweighed confidence voting. In the equation above *λ*_*s*_ are the weights for each individual classifiers.

When unweighted voting is used, all weights are set to 1. When weighted confidence-based voting is utilized, the weights are calculated by choosing the ones that maximize overall classification accuracy in the training dataset, in a cross-validation procedure described in [13]. Mathematically, for a given group of weights *W* = {*λ*_0_, *λ*_1_,…, *λ*_*S*_}, the labeled (segmented) training data is denoted as *R*_train_(*W*), and the ground truth of training data is denoted as *G*_train_. Our goal is to find the optimaset of weights *W*_optimal_ that maximizes the objective classification accuracy function *M* as

(5) $$\begin{array}{c} W_{\text{optimal}}=\underset{W\in R^{S+1}}{\arg\;\max\;}\;M\left(R_{\text{train}}\left(W\right),G_{\text{train}}\right) \end{array},$$

where *M*(·, ·) is a measurement of overall classification accuracy. In this paper, the overall classification accuracy is defined as *A*_correct_/*A*_all_, where *A*_correct_ is the number of correctly classified pixels, and *A*_all_ is the total number of pixel to be classified, both of which can be calculated easily when *R*_train_(*W*) and *G*_train_ are given.

## 3. Performance Evaluation

In this section, we describe both qualitative and quantitative evaluation of the performance of our classification system in three different datasets. We first compare the performances of several voting strategies, as mentioned above. We then test our system on several segmentation tasks and compare the results to those produced by several algorithms selected (and designed) for each application.

With the exception of the aforementioned classification training parameters optimized using cross validation, the parameters pertaining to our algorithm were selected considering the limitations of the available computing power. With the exception of the training set size, all parameters are kept constant throughout all computations in this paper. We set the neighborhood size as 3 × 3 × 3. In addition, for boundary-type pixels, image windows were rotated along the Z axis both clockwise and counterclockwise by 45 degrees. Also, these image windows (including rotated versions) were flipped left and right, up and down in *X*-*Y* plane. For the implementation of multiscale framework, a total of 5 scales (denoted scale 0 to scale 4) were used.

### 3.1. Voting Strategy Comparison

In this subsection, we compare the performances of the voting strategies discussed above. For this experimental evaluation, we chose the IBSR real T1-weighted brain-MR dataset [36], from which it is possible to obtain already segmented (ground truth) tissues such as cerebrospinal fluid (CSF), gray matter (GM), and white matter (WM). In the experiments, four 3D brain images were randomly selected as the training set and one brain image was randomly selected from the rest for testing. In this computation, the training set size contains 1.6 × 10^5^ pixels.

**Figure 4:**
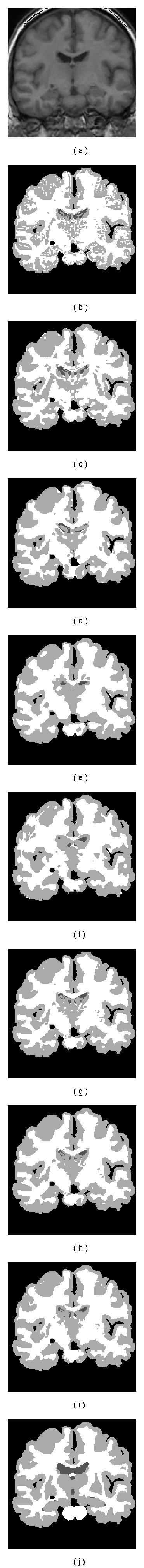
Performances comparison of brain under different cases: (a) original image, (b) result at scale 0, (c) result at scale 1, (d) result at scale 2, (e) result at scale 3, (f) result at scale 4, (g) result of majority voting, (h) result of unweighted confidence voting, (i) result of weighted confidence voting, and (j) ground truth.

Figure 4 shows the segmentation result for one brain. In this figure, the dark gray color represents cerebrospinal fluid (CSF) tissue, light gray represents gray matter (GM) tissue, and white represents white matter (WM) tissue. Part (a) shows the original image, while parts (b)–(f) show the segmentation results by individual classifiers at different scales from 0–4. Parts (g)–(i) show the segmentation results by majority voting (MV), unweighted confidence voting (UCV), and weighted confidence voting (WCV) methods, respectively, while part (j) shows the ground truth information available. Visual interpretation shows that the classification results produced by each individual scale are inferior to results produced by the voting strategies. It is, however, difficult to discern visually which of the voting strategies performs best.

We compare the results quantitatively by using both classification accuracy as well as the dice metric [37]. The dice metric is defined as

(6) $$\begin{array}{c} \frac{2\left|\hat{T_{c}}\cap \tilde{T_{c}}\right|}{\left|\hat{T_{c}}\right|+\left|\tilde{T_{c}}\right|} \end{array},$$

where $\hat{T_{c}}$ denotes the segmented region of pixels for tissue *c*, $\tilde{T_{c}}$ denotes the ground truth set of pixels for tissue *c*, and |·| denotes the set size. As with previous works [15, 19, 38, 39], we focus on segmentation of GM and WM tissues. The overall classification accuracy for each scale as well as for the various voting strategies is shown in Table 1. From this table, it is clear that the weighted confidence voting method is able to achieve higher accuracy than individual classifiers or any other voting methods (on this dataset). We have also performed the same comparison on a histology dataset (described in more detail below) and results were similar. These are omitted here in the interest of brevity. We have chosen to use the weighted confidence voting scheme in the overall assessment of our segmentation algorithm below.

### 3.2. Segmentation of Tissues in Brain MRI Datasets

As mentioned above, segmentation of gray matter tissue (GM) and white matter tissue (WM) from brain-MR images is a popular procedure in biomedical imaging, and several methods have been proposed to this end [15, 19, 38, 39]. We test our system on this application. As above, we use the same brain-MR dataset provided in [36], in which a total of 18 3D MRIs of different brains exist. Each 3D image contains 128 scanned slices. In our test, we use the same 4 brain images selected in the previous section for training, and the remaining 14 brain images are used for testing. The training set contained 1.6 × 10^5^ pixels. To better understand the quality of the results produced by our method, we compare it to the results produced by the method presented in [19], which also utilizes the pixels' intensity neighborhoods as an adaptive nonparametric model of Markov statistics, and produces an optimal classification by iteratively maximizing a mutual-information metric that relies on Markov probability density function.

As in [19], we calculate the mean, median and standard deviation of the dice metric for the WM and GM tissue classes. The results of our method (as well as a summary of the results from [19]) are provided in Table 2. In short, the results indicate that, in general, the accuracies on the target tissues (GM and WM) are similar for both methods.

### 3.3. Segmentation of Tissues in Histology Images

Here, we demonstrate the application of our system to the task of segmenting tissues from histology (H&E stained) images of teratoma derived from human and nonhuman primate embryonic stem cells [40]. Generally speaking, tissue segmentation from histology images of this type is a challenging task due to the complex variation in texture, color, shape, structure, and so forth of the tissues of interest. In addition, teratoma-specific challenges include low intraclass similarity (the same type of tissue often has different visual appearance) and high interclass similarity (multiple types of tissues can have similar visual appearance) [41–43].

To the best of our knowledge, no standard approach for segmenting such images reliably and accurately is known. Since color is an important information in the segmentation of histological images, we test the *K*-means-based color segmentation approach, which has been used in the past to segment similar images [44–46]. The approach usually taken is to convert the color image from *R*∗*G*∗*B* space to *L*∗*a*∗*b* space, and then to use the *K*-means method to cluster pixels in *a*∗*b* color space. Here, we compare our segmentation results to the method described in [44–46]. In order to make the comparison fair, we also utilize the color *a*∗*b* vector together with the same SVM method we use in our system, for the purpose of comparing the performances of different features.

**Figure 5:**
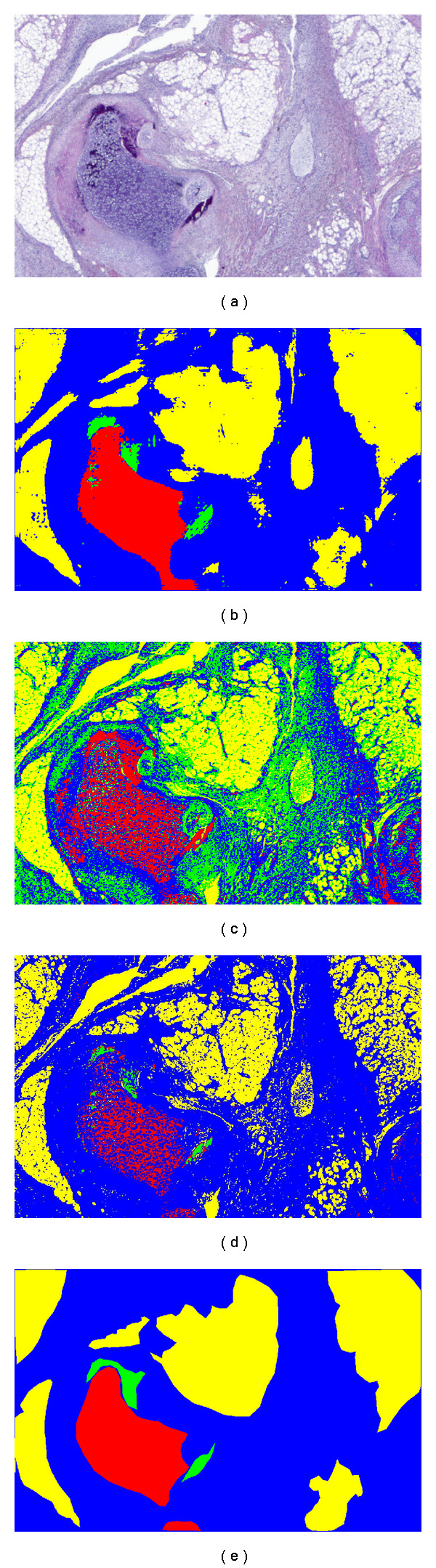
Segmentation of tissues for images of teratoma histology: (a) original image, (b) our result, (c) color *K*-means' result, (d) color SVM's result, (e) ground truth.

The images used in this portion of our validation study were acquired by using a slide scanner to scan the whole sectioned teratoma slices at a high resolution after H&E staining. The color images obtained contain *R*∗*G*∗*B* channels, and the resolutions are 3.527 microns/pixel (0.2834646 pixels/micron) and approximately 3–5 micron thickness (*z*-dimension). Since the number of pixels in raw images is usually large (e.g., size 4824 × 4014), image patches of smaller sizes are randomly cropped (e.g., size from 896 × 932 to 1438 × 1106) for simplification. In this test, 4 images were selected randomly for training, and the remaining 10 images were used for testing. The ground truth segmentations (used in both training and validation) were provided by the pathologist (J.A.O). As before, a 3 × 3 window was chosen for each resolution and the size of training set was chosen to be 1.2 × 10^5^ pixels. In these tests, however, each neighborhood also contained 3 channels corresponding to the *R*∗*G*∗*B* channels of each image.  Figure 5 shows the results of automatic segmentation of four tissue classes: Bone (B), Cartilage (C), Fat (F), and background/other tissues (O). For the approach we proposed, together with the color *K*-means approach and color SVM approach, the green color represents B tissue, red color represents C tissue, yellow color represents F tissue, and blue color represents O tissue regions.

In this application, color represents useful information for classification, although it is clear that a method relying purely on color information cannot perform well in this application since most tissues are heterogeneous in terms of color content. The method we propose, on the other hand, is able to perform reasonably well. To quantify the result, Table 3 reports the overall pixel classification accuracy (a metric also used for quantitative assessment of segmentation of histology images in [4, 11, 47]).

### 3.4. Segmentation of Cell Nuclei from Fluorescence Microscope Images

**Figure 6:**
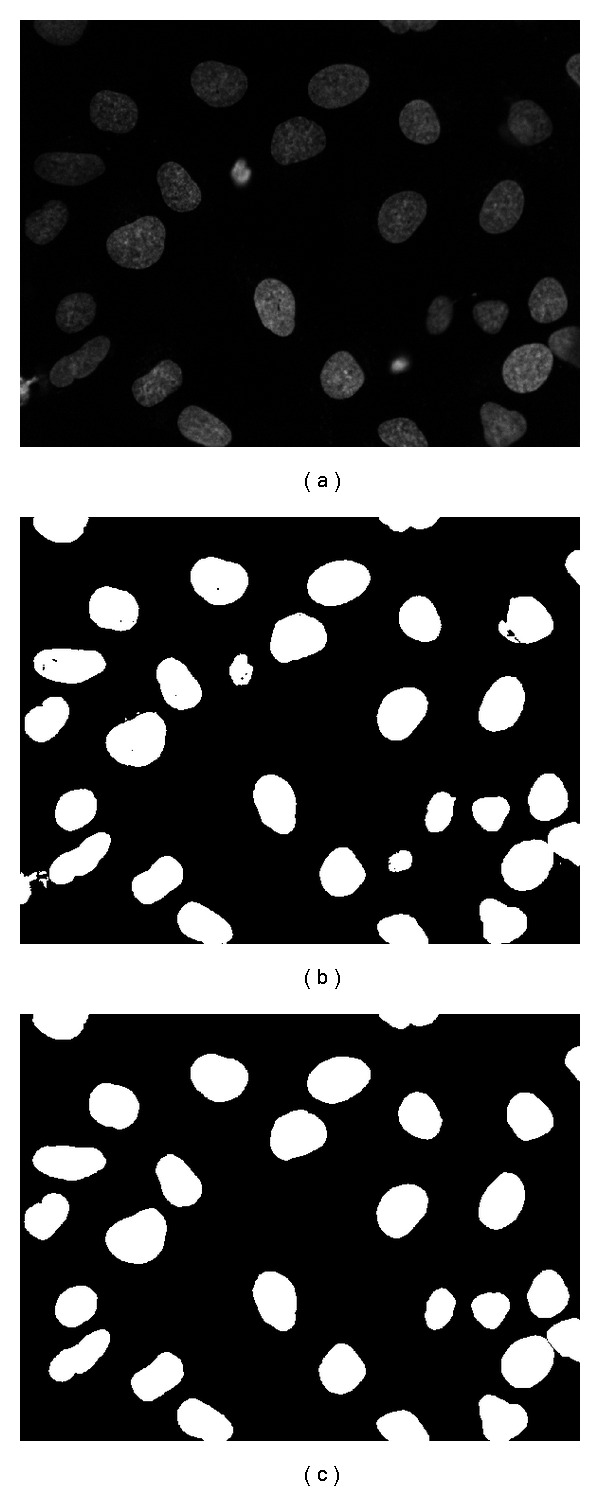
Segmentation of Nuclei: (a) original image, (b) our result, and (c) ground truth.

We test our approach in the task of segmenting nuclei from fluorescence microscope images. The image dataset chosen in this application is available from Dr. Robert Murphy's group at Carnegie Mellon University [48]. The dataset consists of 48 images, out of which 6 images were randomly selected for training and the remaining images were used for testing. We chose the neighborhood window size to be 3 × 3 and the training set size as 8.0 × 10^4^ pixels here. In Figure 6 part (a) a sample image is shown. In part (b) the segmentation result produced by our system and in part (c) the ground truth image (provided by human observers as described in [48]). Visually, the results seem satisfactory, although one may notice small openings in the interior of some nuclei. Such relatively minor artifacts could be easily removed using simple morphological operations (e.g., closing and opening). However, we have purposely refrained from using any postprocessing methods on our results throughout all examples shown in this paper since our main focus is in describing our system and evaluating its performance.

Reference [48] reviews and compares several methods that are commonly used for nuclei segmentation. These methods include: (1) three thresholding-based methods: Ridler and Calvard [49], Otsu [50], and mean pixel value; (2) seeded watershed method [4, 7] operating on a blurred version of the image and the gradient of the image; (3) active masks [51]; and (4) a merging algorithm [52]. Several metrics are used for quantitative evaluation: (1) The Rand and Jaccard Indices: the Rand index (RI) measures the fraction of the pixel pairs where the segmented nuclei and the ground truth agree, which ranges from 0 to 1, which 1 means the perfect agreement. The Jaccard index (JI) is another metric for measuring the fraction of the pixel pairs agreed between the segmented ones and the ground truth. There is no upper bound for the Jaccard index. For both RI and JI metrics, the higher values mean the better segmentation. (2) Spatially-Aware Evaluation Metrics: both the Hausdorff metric and the normalized sum of distances (NSD) metric evaluate the segmented results spatially. For each pixel in the segmented image, its distance to the reference border can be calculated. The Hausdorff metric refers to the largest distance among the pixels which have a disagreement between the segmented objects and the ground truth, and the NSD metric refers to the normalized sum of distances over these pixels. Note that for the NSD metric. 0 means perfect agreement and 1 means no overlap. (3) Error Counting: errors in the segmentation result are counted by comparing each segmented object with the referenced object in the ground truth with which it shares the most pixels. Four classes of errors are counted. Split: two segmented nuclei correspond to the same reference nucleus in the ground truth; merged: two reference nuclei in the ground truth correspond to the same segmented nucleus; added: a segmented nucleus corresponds to the background in the ground truth; and missing: a reference nucleus correspond to the background in the segmented image. More details on these metrics can be seen in [48].

For validation, we compare our results of segmenting the nuclei on the dataset mentioned above to the methods reviewed in [48] using the same metrics, and the quantitative results are shown in Table 4. From the comparison, we can see that although the Hausdorff distance metric and the added error metric of our method is much larger than those of other methods due to the noise effect, which can be easily improved by using some simple morphological operations, the overall results are comparable or better to the results in [48].

## 4. Conclusion and Discussion

We described a supervised learning-based system for segmenting different types of biomedical images. Our focus was to describe a general purpose system that does not require extensive customization to each segmentation application, so long as enough labeled data is available for training. Different from current learning-based methods, which often aim to design specific parametric features for different applications [10, 11], we use intensity neighborhoods as nonparametric feature vectors for pixel classification. Rotations, coordinate inversions, and scales are modeled digitally using standard image processing methods. In addition, a subset sampling strategy based on the *K*-means algorithm is also described. The system is able to handle segmentation of 2D and 3D, as well as scalar and nonscalar (e.g., color) images.

We compared the application of our method to several other segmentation approaches in three distinct biomedical image segmentation tasks: segmentation of tissues from 3D brain MR images, segmentation of tissues in color histology images, and segmentation of nuclei from gray-scale fluorescence microscopy images. We have chosen at least one other relatively modern segmentation method for comparison in each application. Overall, our general purpose segmentation system performed as well as (or at times better than) some of the best available custom tailored methods in each application. We also note that the system we described could be further improved by using other post processing operations such as morphological processing. No post processing operation was used for any of the results presented in this paper.

We note that our system contains several limitations, which offer numerous tasks for future work. An obvious current drawback of our system is that it trades computation time for generality. Let *N* be the window neighborhood size, and *Q*_train_ and *Q*_test_ the numbers of training pixels and testing pixels respectively, for a 2D/3D image segmentation problem, the training computational complexity is *O*(*Q*_train_^3^*N*^2^)/*O*(*Q*_train_^3^*N*^3^), and the testing computational complexity is *O*(*Q*_test_*N*^2^)/*O*(*Q*_test_*N*^3^). In our implementation (all computation times reported were based on a single 2.0 GHz Intel Xeon processor), the computing time for training the SVM classifier for the brain-MR segmentation task (the training set contained 1.6 × 10^5^ samples) was 12.4 hours for one single scale. Segmenting one 3D MR image containing 9.3 × 10^4^ pixels (128 slices) took 3.8 hours. In our histology image segmentation tests, the corresponding computation times were 14.5 hours (1.2 × 10^5^ training samples) for training, and 6.4 hours for segmenting an image of size 1103 × 1421 pixels. Finally, in the nuclear segmentation application, the corresponding computation times were 1.55 hours for training (using 8.0 × 10^4^ samples), while segmenting one single image (size 1030 × 1349) took 0.82 hours.

Another current limitation of our system is related to the selection of the necessary parameters. While some parameters were selected using well-known cross-validation strategies, others (such as the neighborhood window size *N*, the training set size *Q*) were selected based on empirical procedures (related to prior experience) as well as computational complexity considerations. We note again that, with the exception of the number of training pixels, all other parameters were kept constant throughout all experiments in this paper. Given the reasonable accuracies obtained in all experiments, we do not expect the accuracies to change significantly for small changes in these parameters. However, we have not exploited the issue of parameter selection appropriately, and plan to do so in future work.

Yet, another limitation is related to the number of training pixels available for each class (tissue type). We have noticed that when one class contains much fewer pixels than others in the training procedure, the classifier will tend to give low importance to making an error in such class in the testing dataset. An example of this can be seen in the classification of CSF tissue class in brain-MR segmentation (Figure 4), in which the accuracy for classification of CSF tissue is worse than other tissues. Strategies for minimizing such artifacts are also the subject of future studies. We also mention that our method depends on accurately labeled/segmented data. Generally, the labeled data is randomly selected and the amount of training data is selected empirically considering the tissue differences in different modalities of data.

Finally, we predict that the performance of our system could be further improved by fine tuning several of the steps involved in training and classifications. Future work includes investigating different approaches for selecting representative pixels, the appropriate size of pixel neighborhoods, and methods for linking the information from multiple scales, and so forth. We mention that although we have not done this for the results presented in this paper, our general system can also be fine tuned to a given application by selecting application specific intensity normalization procedures as well as using a priori information to select representative pixels.

## Figures and Tables

**Table 1:** Quantitative evaluation for brain MR-data.

Case	Scale 0	Scale 1	Scale 2	Scale 3	Scale 4	MV	UCV	WCV
Overall Accuracy	81.99%	84.77%	84.48%	85.05%	84.09%	88.45%	88.83%	89.11%
GM (dice metric)	0.8411	0.8709	0.8681	0.8744	0.8666	0.9033	0.9070	0.9092
WM (dice metric)	0.8055	0.8277	0.8274	0.8338	0.8161	0.8723	0.8712	0.8806

**Table 2:** Comparison: mean, median, and standard deviation for GM and WM tissues using different methods.

Statistical measure	GM (proposed system/[19])	WM (proposed system/[19])
Mean	0.9053/0.8074	0.8198/0.8868
Median	0.9092/0.8009	0.8382/0.8913
Standard deviation	0.0304/0.0426	0.0676/0.0179

**Table 3:** Quantitative evaluation for images of teratoma histology.

Statistical measure	Bone	Cartilage	Fat	Background/Others
Our Method (accuracy)	59.70%	73.18%	91.09%	88.93%
Color *K*-means (accuracy)	29.79%	51.06%	58.73%	55.20%
Color SVM (accuracy)	27.87%	29.09%	66.16%	68.12%

**Table 4:** Quantitative comparison of nuclei segmentation.

Algorithm	RI	JI	Hausdorff	NSD (×10)	Split	Merged	Added	Missing
AS manual	95%	2.4	9.7	0.5	1.6	1.0	0.8	2.2
RC threshold	92%	2.2	34.8	1.2	1.1	2.4	0.3	5.5
Otsu threshold	92%	2.2	34.9	1.2	1.1	2.4	0.3	5.6
Mean threshold	96%	2.2	26.5	1.0	1.3	3.4	0.9	3.6
Watershed (direct)	91%	1.9	34.9	3.6	13.8	1.2	2.0	3.0
Watershed (gradient)	90%	1.8	34.6	3.0	7.7	2.0	2.0	2.9
Active masks	87%	2.1	148.3	5.5	10.5	2.1	0.4	10.8
Merging algorithm	96%	2.2	12.9	0.7	1.8	2.1	1.0	3.3
Our result	97%	2.5	119.3	0.8	0.8	2.8	3.5	0.3
